# The Association between Dysnatraemia during Hospitalisation and Post-COVID-19 Mental Fatigue

**DOI:** 10.3390/jcm12113702

**Published:** 2023-05-26

**Authors:** Gerardo Salvato, Elvira Inglese, Teresa Fazia, Francesco Crottini, Daniele Crotti, Federica Valentini, Giulio Palmas, Alessandra Bollani, Stefania Basilico, Martina Gandola, Giorgio Gelosa, Davide Gentilini, Luisa Bernardinelli, Andrea Stracciari, Francesco Scaglione, Elio Clemente Agostoni, Gabriella Bottini

**Affiliations:** 1 Department of Brain and Behavioral Sciences, University of Pavia, 27100 Pavia, Italy; 2 Cognitive Neuropsychology Centre, ASST “Grande Ospedale Metropolitano” Niguarda, 20162 Milan, Italy; 3 NeuroMI, Milan Centre for Neuroscience, 20126 Milan, Italy; 4 Department of Laboratory Medicine, ASST “Grande Ospedale Metropolitano” Niguarda, 20162 Milan, Italy; 5 Neurology Department, ASST “Grande Ospedale Metropolitano” Niguarda, 20162 Milan, Italy; 6 Bioinformatics and Statistical Genomic Unit, Istituto Auxologico Italiano IRCCS, 20095 Milan, Italy; 7 Department of Psychology, University of Bologna, 40126 Bologna, Italy; 8 Department of Oncology and Hemato-Oncology, University of Milan, 20122 Milan, Italy

**Keywords:** mental fatigue, long COVID-19, dysnatraemia, electrolyte imbalance

## Abstract

COVID-19 may induce short- and long-term cognitive failures after recovery, but the underlying risk factors are still controversial. Here, we investigated whether (i) the odds of experiencing persistent cognitive failures differ based on the patients’ disease course severity and sex at birth; and (ii) the patients’ electrolytic profile in the acute stage represents a risk factor for persistent cognitive failures. We analysed data from 204 patients suffering from COVID-19 and hospitalised during the first pandemic wave. According to the 7-point WHO-OS scale, their disease course was classified as severe or mild. We investigated the presence of persistent cognitive failures collected after hospital discharge, while electrolyte profiles were collected during hospitalisation. The results showed that females who suffered from a mild course compared to a severe course of COVID-19 had a higher risk of presenting with persistent mental fatigue after recovery. Furthermore, in females who suffered from a mild course of COVID-19, persistent mental fatigue was related to electrolyte imbalance, in terms of both hypo- and hypernatremia, during hospitalisation in the acute phase. These findings have important implications for the clinical management of hospitalised COVID-19 patients. Attention should be paid to potential electrolyte imbalances, mainly in females suffering from mild COVID-19.

## 1. Introduction

Individuals who recovered from Coronavirus disease 2019 (COVID-19) may experience a plethora of persistent symptoms, i.e., ‘long COVID’ [1]. According to the UK National Institute for Health and Care Excellence (NICE) guidelines, the term long COVID describes signs and symptoms that continue or develop after acute COVID-19. It includes ongoing symptomatic COVID-19 (4 to 12 weeks) and post-COVID-19 syndrome (12 weeks or more). These manifestations may include fatigue, muscle weakness, shortness of breath or cough, as well as joint or chest pain [2], implicating multi-organ alterations following the viral infection. Long COVID also includes persistent cognitive difficulties [3,4,5,6,7], and the most frequently described involves attentional impairments [8]. A systematic review performed on 57 studies with 250,351 survivors of COVID-19 found difficulties in concentration (23.8% of the patient sample) [4]. These failures can be present in the form of (mental) fatigue, one of the most experienced persistent symptoms after hospitalisation [9]. Such persistent symptoms impair an individuals’ ability to perform daily activities and affect quality of life [10,11]. 

Our understanding of the factors underpinning persistent cognitive failures after COVID-19 remains limited. Previous studies have suggested a correlation between persistent cognitive failures and disease severity [6,12,13]. Patients who benefited from invasive ventilation presented with a better cognitive status [13]; however, this topic is a current matter of debate. A growing body of research has also shown that sex at birth may play a role. Females are more likely to suffer from persistent symptoms after recovery, with a higher likelihood of reporting persistent fatigue [14,15,16]. This evidence sets the state for challenging scientific investigations, as long COVID mainly affects women [15], although vulnerability and mortality from acute COVID-19 infection are higher in men [17]. Studying the different clinical patterns between males and females during the infection could shed new light on this issue. In this perspective, sex at birth determines different pathological profiles in patients affected by COVID-19. 

A recent systematic review has shown that electrolytic imbalance is prevalent in COVID-19 patients [18]. Interestingly, studies have also highlighted sex differences in electrolyte imbalances caused by SARS-CoV-2 in the acute phase [19]. Moreover, such electrolyte patterns have been associated with COVID-19 disease in hospitalised patients [20]. In patients with COVID-19, SARS-CoV-2 enters the cells using angiotensin-converting enzyme 2 (ACE2) as a receptor, which is one of the main effectors of the brain renin–angiotensin system (RAS). The virus replicates after entry into the cells, and ACE2 gets downregulated. As a result, there is reduced degradation of angiotensin-II, leading to increased aldosterone secretion and a subsequent electrolyte imbalance. This biochemical condition differs between males and females as sex hormones influence the expression and modulation of the brain RAS pathway responses. 

Intriguingly, evidence from other pathologies has shown that dysnatraemia is associated with cognitive failures. For instance, hypernatremia (i.e., sodium levels higher than normal) is associated with cognitive deficits, especially in the elderly [21,22]. Moreover, hyponatremia (i.e., sodium levels lower than normal) may also be associated with adverse cognitive outcomes [23,24]. Specifically, whereas the consequences of acute hyponatremia may be severe, including permanent disability and death, mild and moderate hyponatremia may cause cognitive failures, such as attentional deficits [25,26]. These observations may suggest that the different incidences of persistent cognitive symptoms between males and females who suffered from COVID-19 may be associated with the sex differences in electrolyte imbalances in the acute phase.

Identifying patients at the highest risk is now a research priority to prevent persistent short- and long-term symptoms after recovery. Starting with this clinical and scientific need, the current study aims at exploring whether (i) the probability of experiencing persistent cognitive failures differs on the bases of the disease course severity and the patients’ sex at birth; and (ii) the patients’ electrolytic profile in the acute stage represents a risk factor for persistent cognitive failures. Based on previous findings showing that disease severity plays a role [13], we expected that the odds of presenting cognitive failures would be higher in patients who did not need ventilation therapy in the acute phase (mild COVID-19). Furthermore, as long COVID symptoms are more likely to occur in females [15], we expected that patients’ sex at birth might interact with the disease severity regarding the odds of persistent cognitive difficulties. Lastly, as electrolyte imbalances are one of the recurrent features of COVID-19, which differs between males and females [19], we hypothesised that the odds of cognitive failures could be associated with the electrolyte profile during hospitalisation, particularly in female patients. 

## 2. Materials and Methods

### 2.1. Patients

Inclusion criteria: We collected data from 275 consecutive patients suffering from COVID-19 admitted to the ASST Grande Ospedale Metropolitano Niguarda in Milan during the first pandemic wave in Italy from February to April 2020 (T0). The diagnosis was based on at least one positive test result with the reverse transcriptase–polymerase chain reaction (PCR) for SARS-CoV-2. After two consecutive negative oropharyngeal swabs (i.e., recovery), patients were discharged and followed up via the outpatient service of the same hospital from May to July 2020 (T1). 

**Figure 1:**
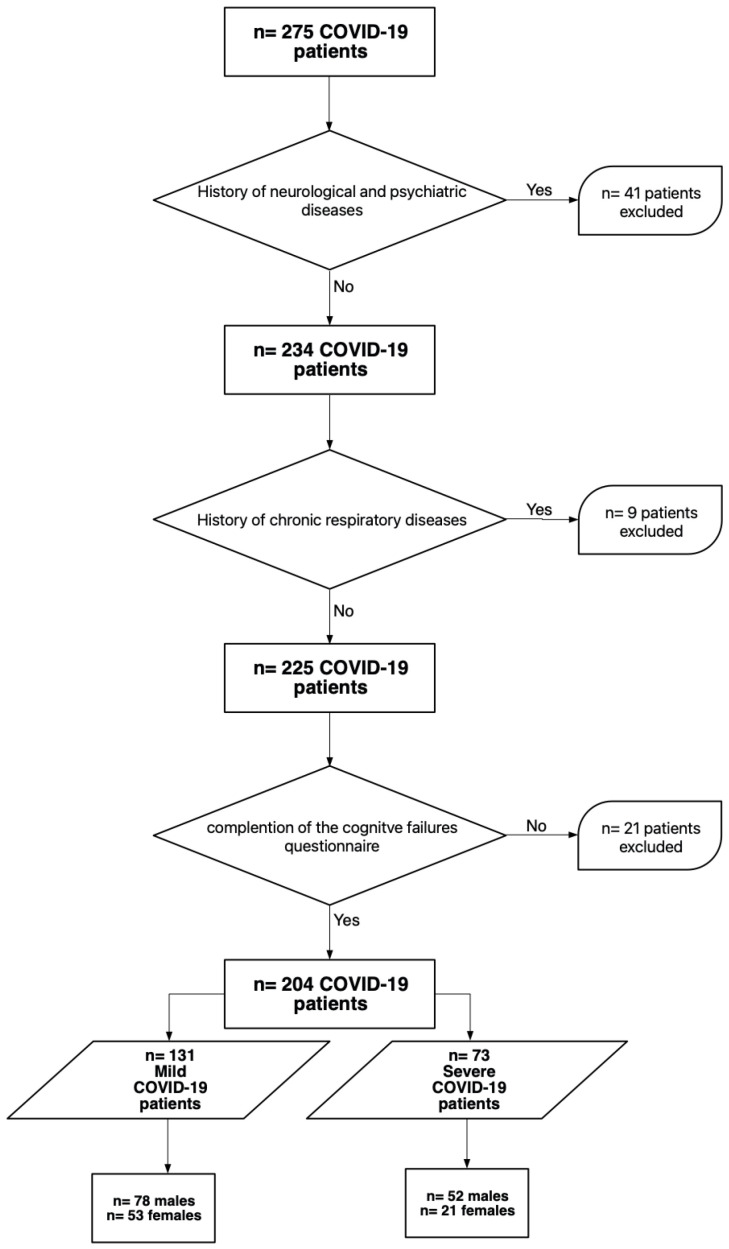
Representation of the patient enrolment workflow of the study.

Exclusion criteria: As this study focuses on subjective cognitive failures, patients with previous neurological and psychiatric disorders were excluded (n = 41). In addition, patients diagnosed with chronic obstructive pulmonary disease (COPD) and obstructive sleep apnoea syndrome (OSAS) were excluded to ensure that any cognitive outcomes were unrelated to previous chronic respiratory illness (n = 9). Furthermore, those patients who did not complete the questionnaire on cognitive failures (n = 21) were not included, and the final sample comprised 204 patients (see Figure 1).

Based on their medical records, patients were classified into two groups according to whether they received orotracheal intubation or CPAP ventilation (ventilated patients), or oxygen therapy or no oxygen therapy at all (non-ventilated patients), thus creating the covariate severity of the COVID-19 course. According to the 7-point WHO-OS scale, the course of COVID-19 in ventilated patients was classified as severe (severe COVID-19), while in non-ventilated patients, it was classified as mild (mild COVID-19). The Ethical Committee Comitato Etico Area 3 Milano approved the study (N92-15032020 and N408-21072020). The study was conducted following the Declaration of Helsinki. Informed consent was obtained from the patients. 

### 2.2. Clinical Questionnaire on Cognitive Failures 

To explore cognitive failures during the health emergency in April 2020, we decided to adopt a clinical tool that could be at the same time effective and quick to administer. Thus, we used a modified version of the Cognitive Failures Questionnaire [27]. We adjusted some of the original questions (such as those involving social interaction) to adapt them to the quarantine situation the patients might have experienced once discharged from the hospital. The questionnaire consisted of 19 statements about possible cognitive failures experienced in everyday life involving several domains, such as attention, memory, gnosis, praxis, orientation in time and space and executive functions (see Table 1 for the complete statements list). Similar questionnaires have also been recently used to test cognitive failures during quarantine/self-isolation for COVID-19 [28]. In our study, patients were asked to indicate the presence or absence of cognitive failures with a “yes/no” response. They could report more than one symptom. At the follow-up visit, the patients came to the Chronicity Service of the ASST “Grande Ospedale Metropolitano” Niguarda, where they underwent a series of assessments throughout the morning. The dedicated healthcare staff administered the Cognitive Failures Questionnaire to all subjects, among other evaluations.

### 2.3. Laboratory Test and Data Sources

We retrospectively extracted the electrolytic profile for each patient from a panel of routine clinical laboratory test results. The tested chemical analytes included chloride (*Cl*^−^), potassium (*K*^+^) and sodium (*Na*^+^). All samples were analysed in duplicate, within one hour from blood collection, using the same analyser and the same lot of reagents. Electrolyte parameters were measured by a Roche Cobas 8000 system (ISE modules). Blood samples were processed in a centrifuge at 3000 rpm (revolutions per minute). The number of samples per patient varied according to clinical practice. Each patient was monitored at least once a day, and a pathological value was confirmed by at least two measurements per protocol. To obtain laboratory variables, a query was created to extract anonymised data using the patients’ ID (a numeric string) from a SQL-based repository in which all analytical results of the tests performed in the laboratory were stored. The fields extracted were sex, date of birth, day of lab tests execution, test IDs, test results and hospital ward. 

### 2.4. Statistical Analysis Plan

Firstly, we compared the demographic variables reported in Table 1 between the patient groups. We performed a chi-square analysis for categorical variables and the t-test or Wilcoxon for continuous ones. Then, a reliability analysis was carried out on the items included in the Cognitive Failures Questionnaire. As the questionnaire involves dichotomously scored items, we used the Kuder–Richardson formula (KR-20), which is a widely used method to evaluate internal consistency in cognitive and personality tests. To explore whether the COVID-19 course severity impacted the odds of experiencing cognitive failure after recovery differently depending on the patient’s sex, a logistic regression model for each item of the questionnaire has been fitted. In each model, disease severity, sex and the interaction between sex and disease severity group were specified as independent variables. We also decided to include the follow-up visit time in the model as it ranged from 3 to 104 days (mean 49.9 (±16.7) days after recovery). Each item representing a distinct cognitive failure was specified as a dependent variable. If the interaction between the course of disease severity and sex was statistically significant, meaning that the effect of COVID-19 course severity on the investigated endpoint was statistically significantly different in the two sexes, we fitted the logistic regression model as above, stratifying by sex to estimate the effect of COVID-19 course severity in each stratum of the sex variable. If the interaction was not statistically significant, a model was fitted such as that described above but with solely the main effects of disease severity group, sex and follow-up visit. 

Lastly, we tested the hypothesis that the odds of presenting specific persistent cognitive failures after recovery could be associated with the electrolyte imbalance observed during hospitalisation. Thus, we fitted logistic regression models considering only the questionnaire items significantly different according to the patient’s sex and disease severity. All the laboratory variables were transformed from continuous into binary variables according to their specific cut-off value: if a variable value was out of the normal range, it was labelled “1”; otherwise, the value was labelled “0”. Based on the literature indicating, for example, that sodium alterations, defined as hyponatremia and hypernatremia, both lead to a poor clinical outcome in patients with COVID-19 [18] and based on the fact that several patients in our study presented with both hypo- and hyper-alteration (see Supplementary Table S2), we decided to generate three single indices (for Na^+^, K^+^ and Cl^−^) that encapsulated electrolyte alteration in both directions. Thus, for each electrolyte, a score equal to 1 means that the variable values are higher or lower than the normal range. For instance, in the case of Na^+^, a score equal to 1 means that patients presented with dysnatraemia (hyponatremia, hypernatremia or both). A normal range for sodium levels in the blood is 135–145 milliequivalents per litre (mEq/L). Levels below 135 mEq/L were considered indicative of hyponatremia, while levels above 145 mEq/L were considered indicative of hypernatremia. For potassium, the normal range is 3.5–5.0 millimoles per liter (mmol/L). Thus, levels below 3.5 mmol/L were considered indicative of hypokalaemia, while levels above 5.5 mmol/L were considered indicative of hyperkalaemia. The normal range of chloride is 98–106 milliequivalents per litre (mEq/L). Thus, levels below 98 mEq/L indicated hypochloraemia, while levels above 106 mEq/L indicated hyperchloremia. Statistical analyses were performed using Jamovi software (version 1.2).

## 3. Results

The final sample comprised 204 patients with a mean age of 57.1 years (±11.9), 130 (63%) of whom were males. The patient’s sex was defined as sex at birth. Our sample demographics align with those of the Italian National Institute of Health, which reported that participants who tested positive during this period were, on average, 58 years old. Patients were assessed at an average of 49.9 (±16.7) days after recovery (follow-up time). Following the classification according to the 7-point WHO-OS scale, the sample included 73 patients who received orotracheal intubation or CPAP ventilation (severe COVID-19 patients), and 131 patients who received oxygen therapy or no oxygen therapy at all (mild COVID-19 patients). The clinical characteristics of the sample separated into the two groups of disease severity are reported in Table 2. The pharmacological treatment was in line with the initial recommendations spread during the first wave of the pandemic and was consistent among patients. For example, 92% of enrolled patients were treated with hydroxychloroquine.

### 3.1. Persistent Cognitive Failures: The Impact of the Disease Course Severity and Sex

Concerning the socio-demographic and clinical variables, we found no significant difference in terms of the interaction between disease course severity and sex for age (*F*_(1,198)_ = 3.3, *p* = 0.069), sex (*X^2^*_(1)_ = 2.8, *p* = 0.096), follow-up time (*F*_(1,188)_ = 0.2, *p* = 0.664) and hospital length stay (*F*_(1,188)_ = 0.8, *p* = 0.373) (see Table 2). Concerning the questionnaire, the reliability analysis of the items showed good internal consistency (*KR-20* = 0.851). 

Results of the logistic regression analysis aimed at investigating whether the odds of cognitive failures may differ on the bases of the patient’s disease severity and sex showed a statistically significant interaction between COVID-19 course severity and sex (*β* = 0.32, *95%CI* [0.08; 0.55]), *p* = 0.009) for mental fatigue (item 18) only. For this item, we performed a subsequent analysis by stratifying by sex. Results of this latter analysis showed a statistically significant effect of group severity in females (*β* = 0.29, *95%CI* [0.06; 0.53], *p* = 0.01), meaning that females who suffered from a mild course compared to a severe course of COVID-19 have a higher risk of presenting with persistent mental fatigue after recovery. No effect was observed in males (*β* = −0.01, *95%CI* [−0.14; 0.11], *p* = 0.82) (see Table 3). 

A logistic regression model, without interaction terms, was fitted for all the remaining items, in which no statistically significant interaction was observed. As reported in Supplementary Table S1, no statistically significant effect of COVID-19 group severity was observed for any investigated item.

### 3.2. Association between Persistent Mental Fatigue and the Electrolyte Profile during Hospitalisation

Due to missing data, we retrospectively analysed laboratory test results from 197 patients (out of 204). The sample was composed of n = 125 mild COVID-19 patients (age: *M* = 58.08 (± 14.5) years; sex: 73 males) and n = 72 severe COVID-19 patients (age: *M* = 57.21 (± 11.9) years; sex: 52 males). For an overview of the distribution of the electrolyte imbalance in our patients, see Supplementary Table S2. The results of the logistic regression models performed on item 18, resulting from the previous analysis, showed a statistically significant risk effect of Na^+^ alteration (*β* = 0.37, *95%CI* [0.09; 0.64], *p* = 0.01) on the odds of presenting with persistent mental fatigue after recovery in females who suffered from a mild course of COVID-19. No statistically significant effects were observed for the remaining electrolytes in females with a mild disease course: *K^+^* (*β* = 0.01, *95%CI* [−0.26; 0.28], *p* = 0.94) and *Cl*− (*β* = −0.04, *95%CI* [−0.33; 0.25], *p* = 0.77).

## 4. Discussion

In Italy, during the first wave of the COVID-19 outbreak in 2020 (21 February–28 June), there was a total of 240,760 confirmed infections with 34,788 deaths. Depending on the disease severity, many symptoms may persist after recovery, mainly affecting women. Among these symptoms, cognitive failures may also be experienced. Little is known about the risk factors underpinning persistent symptoms after recovery. This study set out the challenge to explore whether cognitive failures after recovery depend upon the disease severity, the patient’s sex, and the electrolytic indices during hospitalisation. 

We confirm previous evidence by showing that cognitive failures may persist for approximately one month after recovery. Our findings indicated that the disease severity specifically impacted the attentional system by showing that persistent mental fatigue was higher in patients who suffered from a mild course of COVID-19 (i.e., non-ventilated patients). This result is in line with the study by Alemanno and colleagues [13], in which the authors found a better cognitive status in patients who had undergone invasive (orotracheal) ventilation compared to patients who had undergone non-invasive ventilation or no ventilation at all. The authors reported that 12 out of 22 survivors (54.5%) who underwent orotracheal ventilation one month after hospital discharge showed an impaired total score on the MoCA test, a well-known global cognitive screening test. The same applied to 10 out of the 12 survivors who were treated with non-invasive ventilation (83.3%), 17 out of the 20 survivors who needed oxygen therapy (85%), and 2 out of the 2 survivors who did not need oxygen-based treatment (100%). Furthermore, studies exploring cognitive outcomes in COVID-19 survivors reported mental fatigue as one of the most recurrent symptoms [29,30]. Here, we also showed that patient’s sex plays a role in developing persistent cognitive failures. Indeed, females who recovered from a mild compared to severe course of COVID-19 were more likely to experience persistent mental fatigue. This result aligns with previous evidence highlighting the role of patient’s sex in the presentation of persistent fatigue after recovery. A study on 377 patients has shown that 69% of the sample presented with long COVID. Female sex was independently associated with persistent symptoms, and fatigue was most commonly reported (39.5% of the sample) [16]. Furthermore, another study has shown that females under 50 years reported worse fatigue, with fatigue being more likely in women than in men of the same age [15]. 

**Figure 2:**
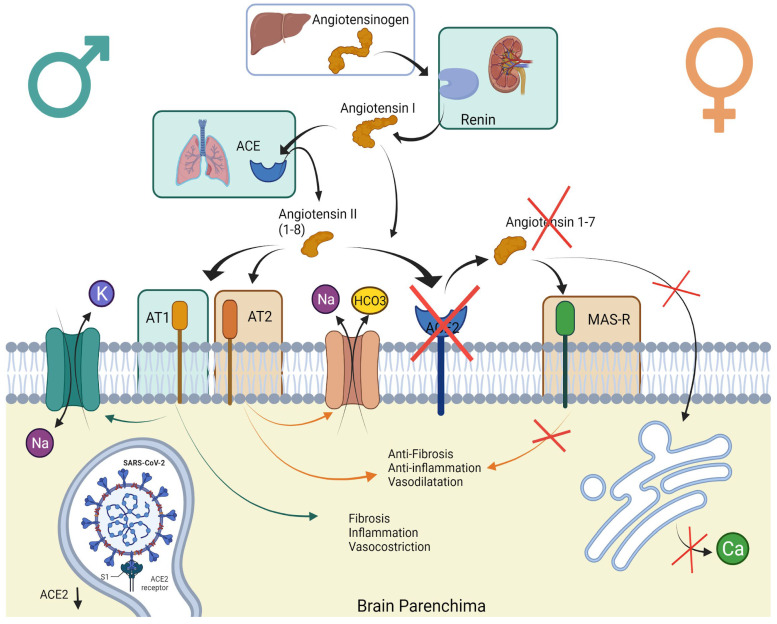
The sex differences in the RAS and response to SARS-CoV-2 injury. Several studies revealed that the AT1 receptor protein is down-regulated by oestrogen, while AT2 receptors are up-regulated. On the other hand, testosterone induced the expression of AT1. AT1 and AT2 receptors have antagonistic actions: Sodium cellular intake is mediated by the AT1 receptor through the increase in Na^+^/K^+^ ATPase activity, while the AT2 receptor activates phospholipase A2, which contributes to activation of the Na/HCO3 symporter system (NBC), mediating sodium cell excretion.

It has been postulated that the aetiology of persistent cognitive failures could be derived from the dual action of the viral infection, which has both direct (immunological and neurological damage) and indirect (hypoxic/respiratory states) consequences [31]. Indeed, two primary routes explain cognition-related deficits in COVID-19 survivors: virus-induced CNS damage (neurotrophic) or non-CNS impairments [32]. Strikingly, we found that in females who suffered from a mild course of COVID-19, persistent mental fatigue was related to an electrolyte imbalance during hospitalisation. The general symptom of fatigue has been previously reported as a consequence of hyponatremia conditions in hospitalised patients [33]. In the case of COVID-19, a possible explanation for the correlation between electrolyte imbalance and mental fatigue in females could be represented by the different sex-dependent expressions of the brain renin–angiotensin system (RAS). In fact, the expression and modulation of the brain RAS pathway responses are influenced by sex hormones. Indeed, with estrogenic stimulation, the ACE2/Ang-(1–7)/MasR system and the ACE2/Ang-(1–8)/AT2 system is increased, while testosterone stimulation mediates the activation of the ACE/Ang-(1–8)/AT1R arm of the RAS. As SARS-CoV-2 enters the cells using ACE2 as a receptor, which is one of the main effectors of the brain RAS, sex differences in the electrolyte imbalance during COVID-19 could be present (see Figure 2).

## 5. Conclusions and Clinical Significance

In summary, this study provided new evidence on the aetiological nature of persistent mental fatigue in COVID-19 survivors who required hospitalisation. In particular, females who suffered from a mild course of COVID-19 had a higher frequency of reporting this symptom one month after hospital discharge. On the one hand, epidemiological studies reported a lower frequency of hospitalisation for females during the COVID-19 infection; on the other hand, electrolyte imbalance occurs more frequently in this population, possibly causing such a persistent cognitive failure. These results pave the way for specific electrolyte rebalancing treatment in hospitalised COVID-19 females who do not require ventilation to prevent cognitive failures after recovery. Therefore, adequate laboratory monitoring and subsequent review of appropriate intravenous water balance medications are important management aspects.

## 6. Limitations

The current study contains some limitations that future investigations may address. Firstly, the nature of the study is retrospective; thus, a prospective case–control study could better define the association between mental fatigue symptoms and the specific direction of sodium imbalance (hypo- or hypernatremia) in hospitalised COVID-19 patients. Moreover, the aetiologies of the changes in sodium concentration could be important contributors to cognitive failures after recovery. Given the association between sodium and fluid balance, sodium may act as a marker for fluid therapies administered during hospitalisation, and future studies may address these issues. Lastly, it would be interesting to correlate the duration of sodium imbalance with the cognitive symptom severity. One of the next challenges could also be to investigate potential factors modulating both the electrolyte and cognitive spheres so that additional confounding factors can be excluded to understand the long-term effects of COVID-19 better.

## Figures and Tables

**Table 1:** Cognitive Failures Questionnaire. The table shows the complete list of statements included in the questionnaire. For each statement, patients provided a yes/no response.

Self-Administered Cognitive Failures Questionnaire
1 Do you find it difficult to remember things that have recently been said or have recently happened?
2 Are you frequently repetitive, that is, do you often say things more than once because you do not remember saying them the first time?
3 Do you often lose things (e.g., glasses) or fail to remember where you put them?
4 Do you have trouble remembering the names of well-known or familiar people?
5 Do you not remember current social events, such as news heard on television or read in the newspaper?
6 Do you confuse one place with another, for example, are you convinced that you are at home even when you are not?
7 Do you get confused about the date, for example, do you make mistakes with the month or year?
8 Do you struggle to find words?
9 Do you frequently say one word instead of another?
10 Do you sometimes say inconsistent things that make it difficult for others to understand what you are expressing?
11 Do you sometimes feel like you do not understand what is being said?
12 Do you have difficulty recognising commonly used objects?
13 Do you have difficultly using familiar objects, for example, household appliances?
14 Do you use objects incorrectly, for example, using a fork to eat soup?
15 Do you often lose the thread of what someone is saying or struggle to follow a conversation?
16 Are you easily distracted by noise or any external stimuli?
17 Do you have difficulty doing two things at the same time, for example, talking while making coffee?
18 Do you get mentally tired easily?
19 When faced with a problem, do you persist with the same behaviour, even if it has proved ineffective several times?

**Table 2:** Demographics and clinical characteristics of the patient samples. The interactions between patient disease severity and sex for the demographic and clinical variables were not statistically significant.

	Mild COVID-19 (N = 131)		Severe COVID-19 (N = 73)		Interaction Disease Severity by Sex
	Males	Females	Males	Females	*p*-Value
*N*	78	53	52	21	0.096
Age, *mean (SD)*	58.3 (14.5)	57.8 (14.3)	55.0 (11.5)	62.4 (11.5)	0.069
Length of hospital stay, *mean (SD)*	15.5 (9.9)	15.01 (7.6)	22.1 (11.1)	24.7 (13.6)	0.373
Follow-up time, *mean (SD)*	53.6 (16.6)	49.6 (13.6)	46.8 (17.4)	45.1 (19.6)	0.664

**Table 3:** Results of the sex-stratified logistic regression model of COVID-19 course severity and cognitive failures, adjusted for the follow-up time.

	Sex = Female			Sex = Male		
ITEM 18	*β*	*95%CI*	*p-Value*	*β*	*95%CI*	*p-Value*
**Mild COVID-19**	0.29	0.06; 0.53	0.01	−0.01	−0.14; 0.11	0.82
**Follow-up time**	0.004	−0.003; 0.01	0.27	0.003	−0.0007; 0.006	0.12

## Data Availability

The data presented in this study are available upon request from the corresponding author.

## Supplementary Materials

The following supporting information can be downloaded at: https://www.mdpi.com/article/10.3390/jcm12113702/s1, Table S1: Results; Table S2: Electrolytics imbalance. Figure S1: Cognitive Failures.
