# Supplementary material for: The Association between Dysnatraemia during Hospitalisation and Post-COVID-19 Mental Fatigue
Source: J Clin Med. 2023 May 26;12(11):3702. doi: 10.3390/jcm12113702 (PMC10253452; doi:10.3390/jcm12113702)
Supplement: Supplementary file 1 [file jcm-12-03702-s001.zip › jcm-2379734-supplementary.pdf]

Supplementary Table S1: **Results.** Results of the logistic regression model for the association between COVID-19 severity group and cognitive failures as investigated by different items. Models were corrected by sex, and follow-up time.

Cognitive failure	β	SE	*p*-value
**ITEM 1**			
COVID-19 group severity (mild)	0.06	0.05	0.24
Sex (female)	0.12	0.05	0.03
Follow-up time	0.001	0.002	0.49
**ITEM 2**			
COVID-19 group severity (mild)	0.05	0.04	0.22
Sex (female)	0.18	0.04	<0.0001
Follow-up time	-0.002	0.001	0.09
**ITEM 3**			
COVID-19 group severity (mild)	0.02	0.06	0.78
Sex (female)	0.13	0.06	0.03
Follow-up time	-0.001	0.002	0.57
**ITEM 4**			
COVID-19 group severity (mild)	-0.03	0.05	0.50
Sex (female)	0.12	0.05	0.02
Follow-up time	-0.01	0.0008	0.57
**ITEM 5**			
COVID-19 group severity (mild)	0.04	0.04	0.35
Sex (female)	0.06	0.04	0.11
Follow-up time	0.0008	0.001	0.46
**ITEM 6**			
COVID-19 group severity (mild)	0.01	0.02	0.36
Sex (female)	0.03	0.02	0.08
Follow-up time	-0.0001	0.0004	0.79
**ITEM 7**			
COVID-19 group severity (mild)	0.07	0.04	0.06
Sex (female)	0.06	0.03	0.10
Follow-up time	-0.0006	0.001	0.57
**ITEM 8**			
COVID-19 group severity (mild)	0.04	0.05	0.44
Sex (female)	0.12	0.05	0.04
Follow-up time	0.003	0.002	0.11
**ITEM 9**			
COVID-19 group severity (mild)	0.02	0.03	0.55
Sex (female)	0.06	0.03	0.09
Follow-up time	0.002	0.001	0.03
**ITEM 10**			
COVID-19 group severity (mild)	0.03	0.02	0.18

Sex (female)	0.05	0.02	0.05
Follow-up time	0.0006	0.0007	0.37
**ITEM 11**			
COVID-19 group severity (mild)	0.04	0.05	0.45
Sex (female)	0.17	0.05	0.0004
Follow-up time	0.002	0.001	0.26
**ITEM 12**			
COVID-19 group severity (mild)	0.009	0.01	0.43
Sex (female)	0.01	0.01	0.25
Follow-up time	-0.0003	0.0003	0.33
**ITEM 13**			
COVID-19 group severity (mild)	0.002	0.02	0.92
Sex (female)	0.02	0.02	0.30
Follow-up time	0.0003	0.0006	0.64
**ITEM 14**			
COVID-19 group severity (mild)	0.009	0.01	0.43
Sex (femal	0.01	0.01	0.25
Follow-up time	-0.0003	0.0003	0.32
**ITEM 15**			
COVID-19 group severity (mild)	0.03	0.04	0.45
Sex (female	0.15	0.04	0.0008
Follow-up time	0.002	0.001	0.14
**ITEM 16**			
COVID-19 group severity (mild)	0.08	0.05	0.15
Sex (female)	0.17	0.05	0.003
Follow-up time	0.0004	0.002	0.82
**ITEM 17**			
COVID-19 group severity (mild)	0.04	0.03	0.21
Sex (female)	0.0009	0.03	0.98
Follow-up time	-0.0009	0.001	0.35
**ITEM 19**			
COVID-19 group severity (mild)	0.03	0.03	0.33
Sex (female	0.03	0.03	0.27
Follow-up time	0.0007	0.0008	0.38

Supplementary Table S2: Electrolytics imbalance. Characteristics of electrolyte alterations in the four patient groups.

Na+

	COVID-19 severity			
		**hypo**	**hyper**	**both**
males	Severe (N=52)	21.2%	36.%	15.4%
	Mild (N=73)	11%	21.9%	1.4%
females	Severe (N=20)	25%	25%	5%
	Mild (N=52)	3.8%	26.9%	1.9%

K+

	COVID-19 severity			
		**hypo**	**hyper**	**both**
male	Severe (N=52)	28.8%	15.4%	15.4%
	Mild (N=73)	12.3%	17.8%	6.8%
female	Severe (N=20)	55%	10%	15%
	Mild (N=52)	25%	11.5%	1.9%

Cl-

	COVID-19 severity			
		**hypo**	**hyper**	**both**
male	Severe (N=52)	36.5%	1.9%	0%
	Mild (N=73)	24.7%	4.1%	0%
female	Severe (N=20)	40%	0%	5%
	Mild (N=52)	23.1%	5.8%	0%

Supplementary Figure S1: **Cognitive Failures**. Percentage of cognitive failures reported in the four groups.

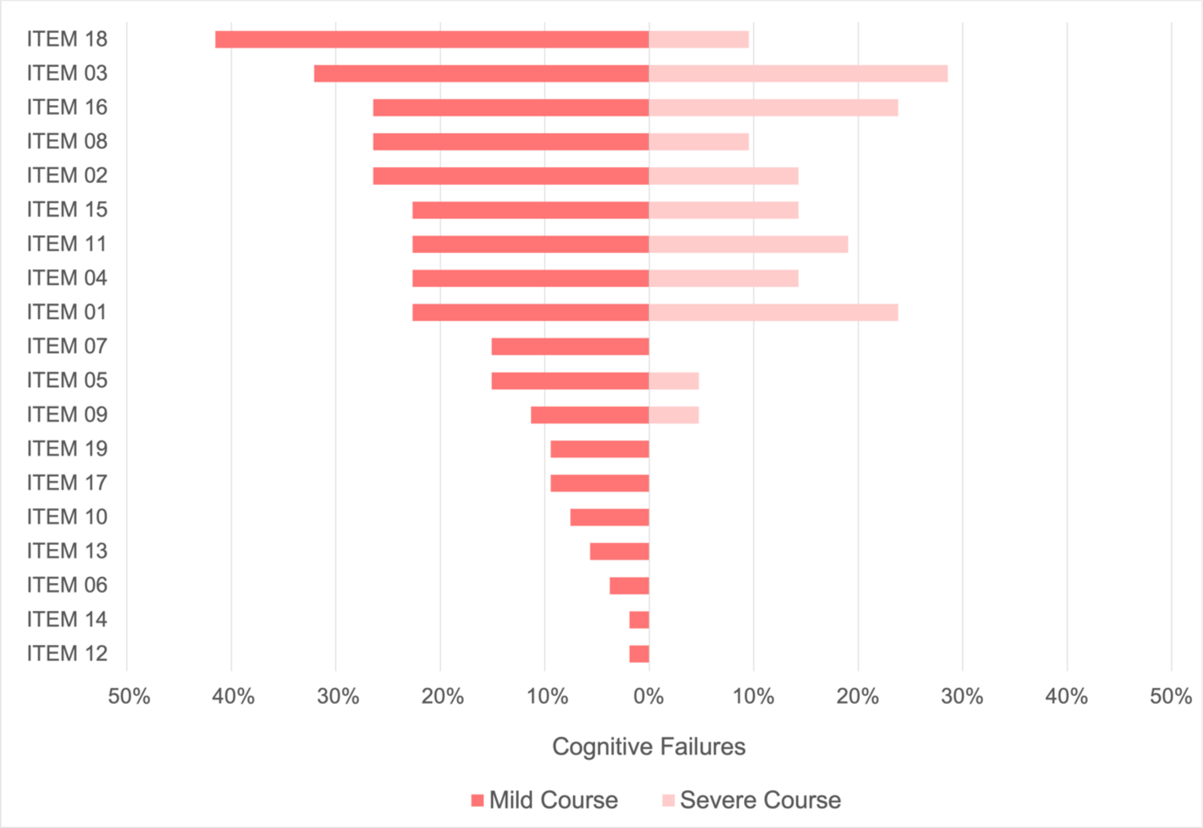

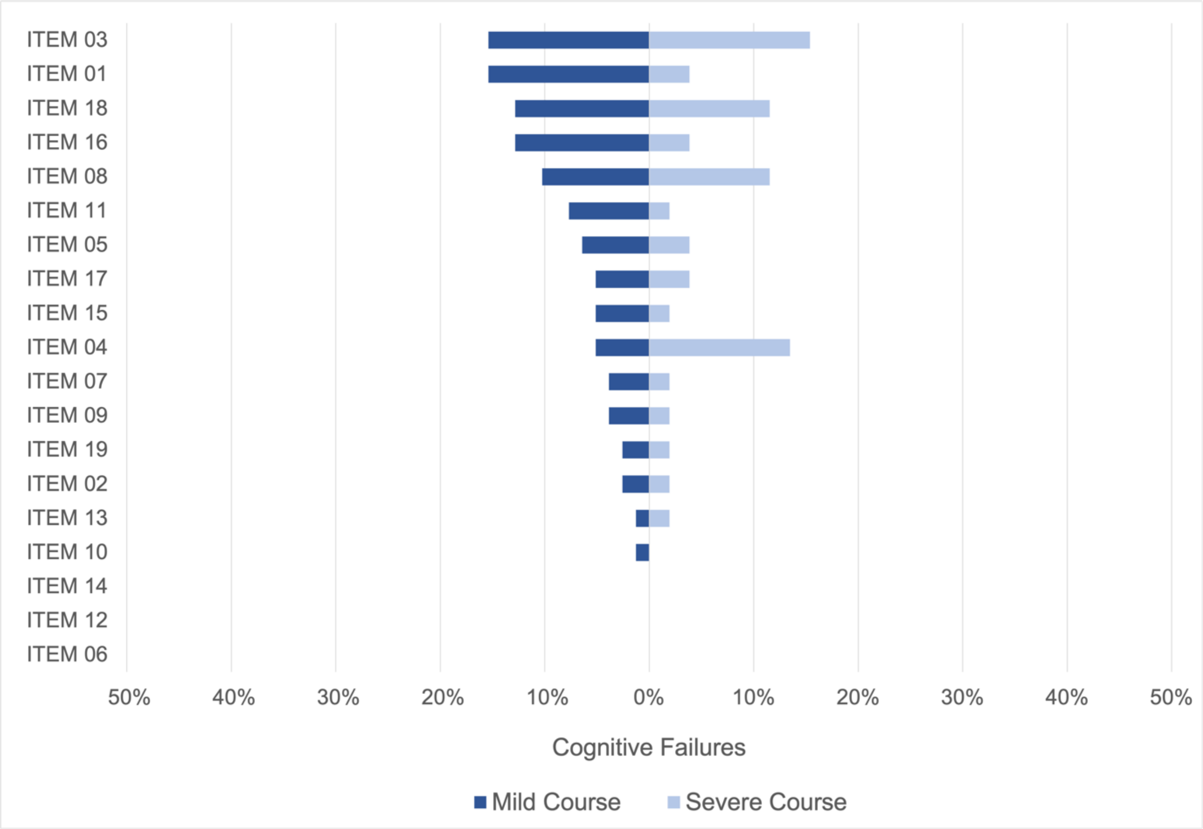
ITEM 03
ITEM 01
ITEM 18
ITEM 16
ITEM 08
ITEM 11
ITEM 05
ITEM 17
ITEM 15
ITEM 04
ITEM 07
ITEM 09
ITEM 19
ITEM 02
ITEM 13
ITEM 10
ITEM 14
ITEM 12
ITEM 06
50% 40% 30% 20% 10% 0% 10% 20% 30% 40% 50%
Cognitive Failures
Mild Course
Severe Course
